# The functional landscape of Golgi membrane protein 1 (GOLM1) phosphoproteome reveal GOLM1 regulating P53 that promotes malignancy

**DOI:** 10.1038/s41420-021-00422-2

**Published:** 2021-03-01

**Authors:** Qi Song, Xiang He, Ying Xiong, Junlan Wang, Lei Zhang, Elaine Lai-Han Leung, Guoping Li

**Affiliations:** 1 Laboratory of Allergy and Precision Medicine, Chengdu Institute of Respiratory Health, The Third People’s Hospital of Chengdu, Chengdu, Sichuan China; 2 State Key Laboratory of Quality Research in Chinese Medicine, Macau University of Science and Technology, Taipa, Macau(SAR) China; 3 Department of Cardiothoracic Surgery, The Affiliated Hospital, Southwest Medical University, No. 25, Taiping St., Luzhou, Sichuan China; 4 Department of Pulmonary and Critical Care Medicine, Chengdu Third People’s Hospital Branch of National Clinical Research Centre for Respiratory Disease, Affiliated Hospital of Chongqing Medical University, Chengdu, Sichuan China; 5 Department of Pulmonary and Critical Care Medicine, Hospital of Sichuan Friendships, Chengdu, Sichuan China

**Keywords:** Non-small-cell lung cancer, Non-small-cell lung cancer

## Abstract

Golgi membrane protein 1 (GOLM1) was implicated in carcinogenesis of multiple types of cancer. However, Phosphoproteome landscapes of GOLM1 overexpression in lung cancer remain largely unknown. In this study, using data from the Cancer Genome Atlas (TCGA) and phosphoproteome, we systematically evaluated the feature of GOLM1 and studied its prognostic value in non-small cell lung cancer (NSCLC). The proliferation, migration, and invasion capacities in PC9 cell with GOLM1 overexpression were determined using Trans-well system assay. Tumor engrafts was visualized in mice models and confirmed by ex vivo. An increased expression of GOLM1 had shorter overall survival (OS) in patients with NSCLC in TCGA database. GOLM1 in single gene set enrichment analysis (GSEA) related to adherent’s junction, cell cycle, and pathway in cancer. Overexpression of GOLM1 in GOLM1OE PC9 cells promoted cell proliferation, migration, and invasion. Decreased migration and invasion potential were also observed in knockdown of GOLM1 in GOLM1KD PC9 cells in migration assay. An increased expression of GOLM1 could significantly increase the growth of tumor in xenograft mice models. phosphoproteome analysis showed 239 upregulated and 331 downregulated Phosphorylated proteins in GOLM1OE PC9 cells. Overexpression of GOLM1 in GSEA was significantly related to P53 in MAPK signaling pathway. Overexpression of GOLM1enhanced the phosphorylation of P53 protein at site S315 but inhibited the formation of P53 tetramers. These results indicate that overexpression GOLM1 enhances non-small-cell carcinoma aggressiveness through inhibited the formation of P53 tetramer.

## Introduction

Across the globe, regardless of cancer morbidity or mortality, lung cancer always ranks first among malignant tumors. Prediction based on reliable data, there will be 2.1 million new cases of lung cancer and 1.8 million deaths in 2018, which will be close to one-fifth (18.4%) of cancer deaths^1^. According to the latest U.S. data statistics, the downward trend in the incidence of lung cancer continue to maintain in U.S., which should be attributed to sound screening programs and efficient multiple prevention strategies^2^. The U.S. experience shows that prevention and screening programs can effectively reduce the incidence of lung cancer. Screening can detect preclinical abnormalities before clinical manifestations occur, following by targeted intervention before or at the early stage cancer, when therapies are still effective^3^. Many scientific research and effective control activities show that nearly three fifths of cancer deaths can be avoid by reducing exposure to recognized cancer risk factors^4^. Half of cancers can be prevented^5^ and one-third of cancers can be cured if they are diagnosed early. Biomarkers that make up the most important part of screening have long been used for detecting premalignant lesions or early invasive disease^6^.

It is well established that there is no effective early diagnostic biomarker for lung cancer. To find new biomarkers that play an active role in early detection of cancer has become a major research hotspot. Through the hard work of large number of candidates’ screening, Golgi membrane protein 1 (GOLM1) was identified as a potential biomarker for lung cancer^7^. Correspondingly encode protein of GOLM1 is a type II Golgi transmembrane protein which also named Golgi phosphoprotein 2 or Golgi protein 73^8^. The expression of this encoded protein has been observed to be upregulated in lung cancer tissues^9,10^. As one component of the Golgi complex, which mediates sorting and modification of cargo proteins exported from the endoplasmic reticulum^11^, GOLM1 plays a key role in protein biosynthesis in the rough endoplasmic reticulum and protein transportation through the Golgi apparatus. Abnormal overexpression of GOLM1 correlates with many kinds of cancers and diseases, such as liver cancer and in prostate cancer tissue^7,12,13^. Therefore, GOLM1 has the potential to become a new tumor biomarker.

Nowadays, little is known about biological functions and molecular mechanisms of GOLM1 in cancer. Several studies have indicated that GOLM1 plays a crucial role in promoting tumor cell invasion, migration, growth, and metastasis in liver and prostate cancers^12,13^. One study revealed that GOLM1 acts as an oncogene promoting liver cancer by adjusting epidermal growth factor receptor/receptor tyrosine kinase (EGFR/RTK) cell-surface recycling. Meanwhile, a study observed GOLPH2 expression was markedly upregulated in human lung adenocarcinoma tissue^7^. Subsequently, occasional studies have discussed the relationship between GOLM1 and lung cancer^14–16^. However, more studies are needed to reveal the role of GOLM1 in lung cancer.

Through data mining in TCGA, we found that GOLM1 is associated with lung cancer, especially lung adenocarcinoma, and the expression level of GOLM1 is causally related to overall survival. In our research, we confirmed the conclusion drawn by previous studies that GOLM1 is overexpressed in lung cancer. Upregulation of GOLM1 markedly improved cell proliferation, migration, and invasion in the lung cancer cell line, PC9, and PC9 xenografts in nude mice. According to our phosphoproteome-based kinase activity profiling, GOLM1 exercises its tumor-promoting function through many signaling pathways. Consequently, GOLM1 may be used as a stable biomarker for diagnosing and as follow-up indicator of therapeutic effects, as well as a therapeutic target in lung cancer.

## Materials and methods

### Patient samples

Fresh normal, adjacent and cancer tissues from eight patients with lung cancer (lung adenocarcinoma was confirmed by pathological examination after surgical resection) for western blot and immunohistochemistry (IHC) analyses were obtained from the Department of Pathology of Affiliated Hospital of South West Medical University (SWMU, Luzhou, Sichuan, China). The experiments involving clinical samples were approved by the Research Ethics Committee, Affiliated Hospital of SWMU. The informed consent of the patients was acquired before the collection.

### Cell lines and culture reagents

The human lung cancer cell line, PC9, was purchased from the Zhong Qiao Xin Zhou Biotechnology Co., Ltd. (Shanghai, China). The cell lines were acquired certificate of STR in 2017 and maintained in RPMI 1640 (Gibco/Thermo Fisher, Scoresby, Australia). Medium were supplemented with 10% fetal bovine serum (Gibco, Grand Island, NY). Cell lines were cultured in incubators (5% CO_2_, 37 °C).

### Cell viability assay

The cells were seeded into 96-well plates at a density of 5 × 10^3^ per well and allowed to grow overnight. PFT-α was dissolved in DMSO and diluted with DMEM medium to the final concentrations of 40 μmol. Nutlin3 was dissolved in DMSO and diluted with DMEM medium to the final concentrations of 10 μmol. The tumor cells were incubated with PFT-α/Nutlin3 for 24 h before the CCK-8 assay (Biosharp, China). Absorbance values were measured at 450 nm and survival curves were plotted using GraphPad software.

### Vector construction and lentivirus packaging

The GOLM1 cloning of coding sequence (CDS) was amplified by polymerase chain reaction using primers: GOLM1-BamH IF: 5′-CGCGGATCCATGATGGGCTTGGGAAAC-3′, GOLM1-Not I-R:5′-ATTTGCGGCCGC GAGTGTATG ATT CCGCTTTTCA-3′, and cloned into a pLEX-MCS vector (Thermo Scientific, Waltham, MA, USA) with restriction enzymes BamH I and Not I (New England Biolabs, Ipswich, MA, USA). Lentivirus were packaged through pLEX-MCS, VSVG, and △8.9 co-transfect HEK-293T cells by Lipo 2000 (Thermo Scientific). The stable PC9 cell lines, GOLM1KD (GOLM1 knockdown) and GOLM1OE (GOLM1-overexpressing), were obtained by lentiviral infection and puromycin (1 µg/mL) screening. Lentiviral vector containing a non-targeting scramble RNA was used as a negative control (empty vector).

### Immunohistochemistry

Section and staining were performed by the Department of Pathology of the Affiliated Hospital of SWMU. IHC was performed according to standard procedures. The primary antibody, anti-GOLPH2 (also named GOLM1, 1:1000), were used for staining. Results were obtained by digital slice scanner (Hamamatsu Photonics, Hamamatsu, Japan). An immunoreactivity score system was hired to evaluate the expression of GOLM1 (see Supplementary Data A).

### Cell-cycle analysis

Briefly, cells were cultured on cover slips and were fixed in 4% paraformaldehyde for 20 min. After permeabilization with 0.2% Triton X-100 (Sigma) for 10 min, the samples were blocked with 2% bovine serum albumin for 1 h. Then, samples were stained with 4′,6-diamidino-2-phenylindole (DAPI, Solarbio, C0065, China) and TRITC phalloidin (1:200, Solarbio, CA1610, China) to label the nuclei and cytoskeleton, respectively. Imagess were acquired by the scanR system on the Olympus IXplore SpinSR super resolution microscope (Olympus, Germany) and analyzed employing cellSens software (Olympus, Germany).

### Western blot analysis

Lytic samples were run on 8–10% sodium dodecyl sulfate-polyacrylamide gel electrophoresis (PAGE) and 8% native-PAGE, transferred to polyvinylidene fluoride membranes and probed with anti-GOLPH2 (1:1000, Abcam, ab109628), anti-P53 (1:1000, CST, 2524S), anti-p-P53 (Ser 315) (1:1000, Bioss, bs-3704R), anti-GAPDH (1:1000, Abkine, A01020), and anti-β-actin (1:500, Genscript, A00730). After incubating with anti-rabbit or anti-mouse IgG secondary antibodies membranes were conjugated to horseradish peroxidase at a dilution of 1:5000 and visualized with an enhanced chemiluminescence system (Bio-Rad, Hercules, CA, USA).

### Wound healing assay

Incubated cells overnight in serum-free RPMI 1640 medium. Create a gap without cells of 500 µm width and replaced the medium with serum-containing medium. After incubated in incubator for 24 h, photographed the areas of cell migration using microscope (Olympus, Tokyo, Japan). The images were analyzed combining Image J and GraphPad Prism software (version 6.0; GraphPad Software, Inc., La Jolla, CA, USA)

#### Transwell cell invasion and migration assay

Precoated Transwell inserts (Corning, New York, NY, USA) with 20 µg/mL Matrigel (Corning, New York, NY, USA) dilute with RPMI-1640 medium. Incubated cells overnight in serum-free RPMI 1640 medium, then seeded 5 × 10^5^ cells resuspending with serum-free medium in each precoated transwell insert; filled serum-containing medium in the culture dishes for adaptation. After incubated in incubator for 24 h, fixed and stained the cells on the lower side of the insert filter. Photographs were taken in five random views through microscope. Image processing and analysis were performed by Image J and GraphPad Prism software.

### Xenograft mouse model

Totally, 1 × 10^7^ cells were injected subcutaneously into left armpit of the 5-week-old female BALB/c nude mice (21 nude mice were randomly divided into three groups of seven in each group by computer random number method). Measurements were performed every 5 days. Tumor volumes were calculated using a standard formula: tumor volume (mm^3^) = width (mm^2^) × length (mm) × 0.5. All animal experiments were approved by the Animal Ethics Committee of SWMU.

### Protein extraction and trypsin digestion

Protein samples of cells were acquired by dissolving in protein lysis buffer (8 M urea, 1% Protease Inhibitor Cocktail) and homogenizing in an ultrasonic processor (Scientz, Ningbo, China). The supernatant was collected after centrifugation at 12,000×*g* and 4 °C for 10 min.

Tandem Mass Tags/isobaric Tagging for Relative and Absolute Quantification (TMT/iTRAQ) Labeling

Peptides acquiring from digestion went through desalting by Strata X C18 SPE column and vacuum-drying. After reconstituted in 0.5 M TEAB, peptides were processed as the protocol for TMT kit/iTRAQ kit (Thermo Fisher Scientific Inc.) providing by the manufacturer

### HPLC fractionation

HPLC was hired to fractionate peptides into fractions using a Thermo Betasil C18 column (5 μm particles, 10 mm ID, 250 mm length).

### LC–MS/MS analysis

Samples were loaded on an ultra-high-pressure liquid chromatography (UPLC) system (EASY-nLC 1000, Thermo Fisher Scientific). The peptides were separated on a 15-cm self-made column (inner diameter was 75 μm) at 60 °C with a constant flow rate of 400 nL/min from 6 to 23% acetonitrile in 0.1% formic acid over 26 min, 23 to 35% in 8 min, followed by climbing to 80% acetonitrile in 0.1% formic acid in 3 min for column washing and then holding 12 min in 80% acetonitrile in 0.1% formic acid. A nano-spray ion (NSI) source was hired to introduce the peptides into the mass spectrometer. Then an orbitrap elite mass spectrometer (Q ExactiveTM Plus, Thermo Fisher Scientific) was employed. One full MS scan was performed at the m/z scan range 350–1800, and with a resolution of 70,000 in the Orbitrap. Twenty MS/MS scans (automatic gain control was set at 50,000) with a 15.0 s dynamic exclusion were performed using a data-dependent procedure alternating between each MS scan.

### Affinity enrichment

Mixtures of peptides were incubated with an immobilized Metal Affinity Column (IMAC) microsphere (GEHealthcare, Waukesha, WI, USA) suspension with vibration in loading buffer (50% acetonitrile/6% trifluoroacetic acid) at first. After centrifugation, sediment containing the IMAC microspheres with enriched phosphopeptides was saved and the supernatant was removed. Followed by washing with 50% acetonitrile/6% trifluoroacetic acid and 30% acetonitrile/0.1% trifluoroacetic acid sequentially the nonspecifically adsorbed peptides were removed from the IMAC microspheres. Through vibration with the elution buffer containing 10% NH_4_OH, the enriched phosphopeptides were eluted from the IMAC microspheres. After the collection of the phosphopeptides dissolving in the supernatant, lyophilization was accomplished for next LC–MS/MS analysis.

### Database search

The raw MS/MS data were processed through MaxQuant software (version 1.5.2.8). Tandem mass spectra were searched against the UniProt human database. In initial search 20-ppm mass tolerance for precursor ions was set and 5-ppm using in main search. Trypsin was selected as the protease requiring at least six of peptide length and with maximum four missing cleavages. FDR less than 1% was set at peptide.

#### The functional enrichment analysis of the gene ontology/Kyoto Encyclopedia of Genes and Genomes pathway

The files of Gene Ontology (GO) database among proteome were downloaded from the UniProt-GOA database (http://www.ebi.ac.uk/GOA/). After matching the enriched protein ID to UniProt ID, annotation was mapped identified protein IDs by GO IDs. Then classification was performed through GO annotation based on three categories: biological process, cellular component and molecular function. The category of GO with a corrected *p* value < 0.05 was considered significant. To annotate protein pathway, Kyoto Encyclopedia of Genes and Genomes (KEGG) database was employed. Protein’s KEGG database description was annotated by KAAS (KEGG online service tools). The results of annotation were mapped on the KEGG pathway database by KEGG mapper (KEGG online service tools). The pathway of KEGG with a corrected *p* value < 0.05 was considered significant.

### Statistical analysis

SPSS (versions 19, IBM SPSS Inc.; Chicago, IL, USA) and GraphPad Prism6 software Statistical analyses were hired for statistical analysis. All the experiments were repeated three times. One-way analysis of variance was employed to compare continuous variables between more than two groups. An independent Student’s *t* test was used for comparing continuous variables between two groups. Statistical significance between values was determined by *p* value (*p* value < 0.05 was considered significant).

### Micro PET/CT

Anesthetized nude mice were disinfected with 75% alcohol and then inoculated with 200 μL of one of the two cell suspensions in the middle of the right armpit. The mice were disinfected again and placed in laminar air flow rack while their physical signs were monitored. Two weeks after grafting, the mice were scanned to detect the size of tumor nodes and to calculate the maximum Standardized Uptake Value (SUVmax) of tumor nodes. Briefly, before scan all mice were fasted for 12 h. After anesthetized with 2% isoflurane, the mice were injected with 18F-FDG (200 lCi each) through intraperitoneal injection. Scans were performed 1 h later after 18F-FDG injection. Imaging data including transaxial, coronal, and sagittal reconstructions was acquired for interpretation. Then, the mice were placed in laminar air flow rack while their physical signs were monitored till death.

## Results

### GOLM1 expression, prognostic association, and GSEA in TCGA database

**Fig. 1:**
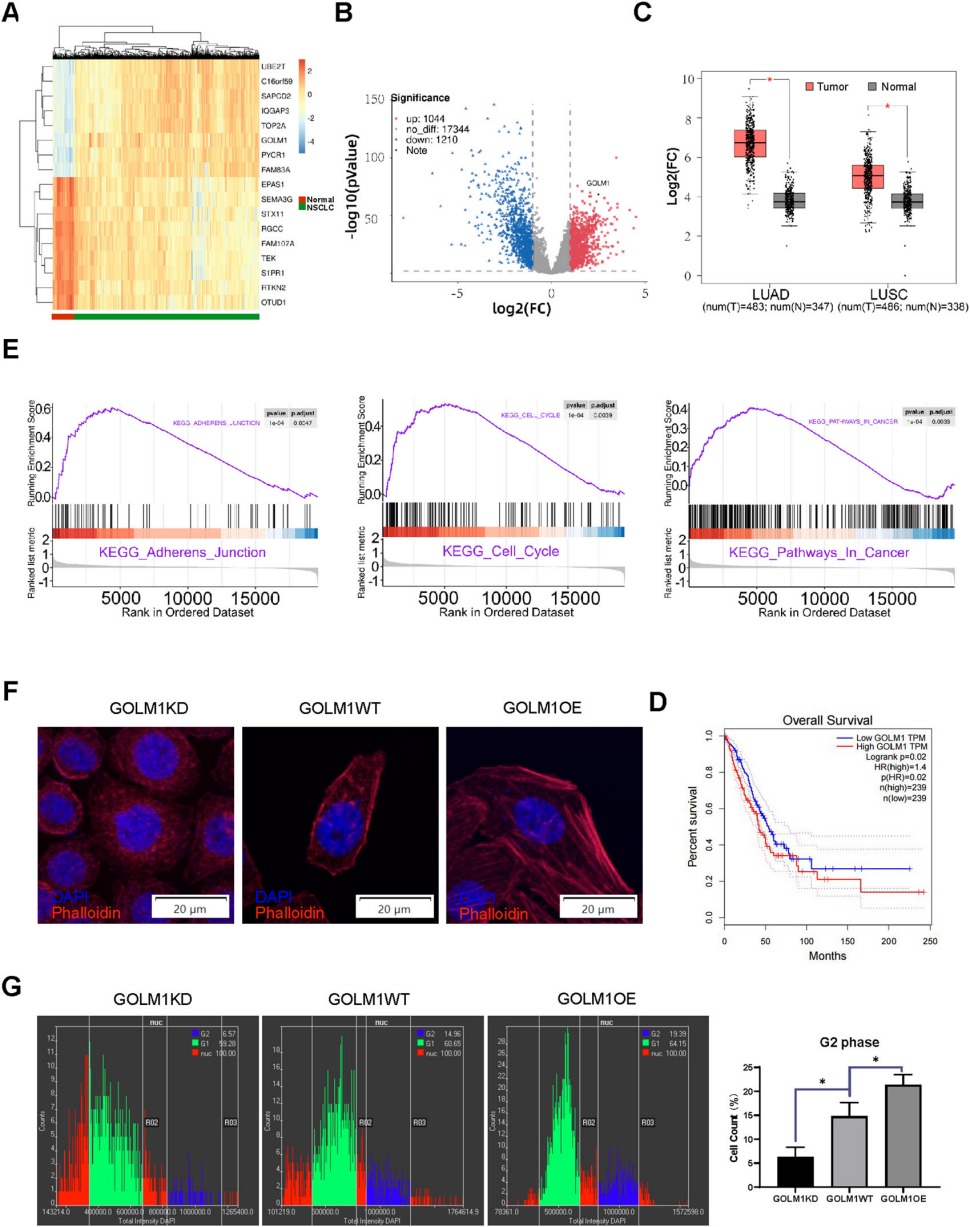
GOLM1 is highly expressed in patients with non-small cell lung cancer whose expression level is negatively correlated with patients’ OS. **A** Heatmap of expression levels of suspect candidate differentially expressed genes (DEGs) in NSCLC patients from TCGA dataset. **B** The DEGs volcano map in TCGA dataset (GOLM1 was marked). **C** Expression levels of GOLM1 in lung adenocarcinoma (LUAD) and squamous cell lung carcinoma (LUSC) from TCGA dataset. Pink refers to tumor group, Grey refers to normal group. (Data were means ± SEM. *P < 0.05). **D** Overall survival (OS) of the lung adenocarcinoma patients with GOLM1 high expressing level against low expressing level. **E** Single gene GSEA analysis of KEGG reference gene sets for GOLM1 high expression groups against low expression groups from TCGA dataset. **F** Actin polymerization was assessed through immunofluorescence assays. Red fluorescence represents Phalloidin, and blue (DAPI) represents cell nucleus. **G** The cell-cycle assay through high content screening (HCS) system and the statistical analysis of cell count (%) in G2 phase among GOLM1KD, GOLM1WT, and GOLM1OE group. GOLM1KD = GOLM1 knockdown group, GOLM1WT = wild-type group, GOLM1OE = GOLM1 overexpression group. Data were means ± SEM. *P < 0.05, *n* = 3.

The dataset from TCGA database contains 533 patients with NSCLC and 59 normal patients were downloaded, collated, and analyzed. All data were normalized and processed with R language packages “TCGAbiolinks”. We determine the aberrantly expressed genes through the differential expression analysis in NSCLC (|Log2 fold change| > 1, adjusted *p*.value < 0.05). We finally selected top seven genes with abnormally high expression (C16orf59, GOLM1, IQGAP3, PYCR1, SAPCD2, TOP2A, and UBE2T) and top nine genes with abnormally low expression (EPAS1, SEMA3G, STX11, RGCC, FAM107A, TEK, S1PR1, RTKN2, and OTUD1) in NSCLC whose expression level were closely related to patient OS (Fig. 1A, B and Supplementary Data B). In order to confirm the expression of these genes in the tumor tissues of real clinical lung cancer patients, we finally determined GOLM1 as the study object. Then, GEPIA (gene expression profiling interactive analysis) database was hired for bioinformatics prediction. We found that GOLM1 expression in both lung adenocarcinoma and lung squamous cell carcinoma was significantly higher than that in the normal control group, and the different level in lung adenocarcinoma was statistically significantly higher than that in lung squamous cell carcinoma (Fig. 1C). We looked at the GOLM1 expression in patients and found high GOLM1 expression correlated with decreased OS (Fig. 1D). To further explore the role of GOLM1 in the organism, we extracted the expression profiles of 59 healthy non-smokers from TCGA database and divided them into two groups: high and low-expression level of GOLM1. We performed GSEA and found that the expression of GOLM1 was mainly associated with malignancies, including “adherents junction”, “cell cycle”, “pathway in cancer” (Fig. 1E), which inferred that GOLM1 may play an important role in the course of malignant tumors. Next, we explored the cytoskeleton rearrangements through immunofluorescence assays in GOLM1 manipulative PC9 cell lines. We found that overexpression of GOLM1 increased the polymerization of actin, which were evaluated by microscopic observation after TRITC-phalloidin staining. Meantime, GOLM1 knockdown showed a decrease in the polymerization of actin (Fig. 1F). To investigate whether GOLM1 regulated the cell cycle of tumor cells, imaging data were processed by the ScanR Analysis-software to determine Cell Cycle through DNA content quantitation assay. As shown in Fig. 1G, GOLM1 downregulating led to a significant decrease in the percentage of cells in G2 phase compared to GOLM1WT cells. On the other hand, the percentage of cells in G2 phase was significantly increased in GOLM1 overexpressing cells compared to WT cells (Fig. 1G).

### Lung cancer showed high GOLM1 expression in clinic sample

**Fig. 2:**
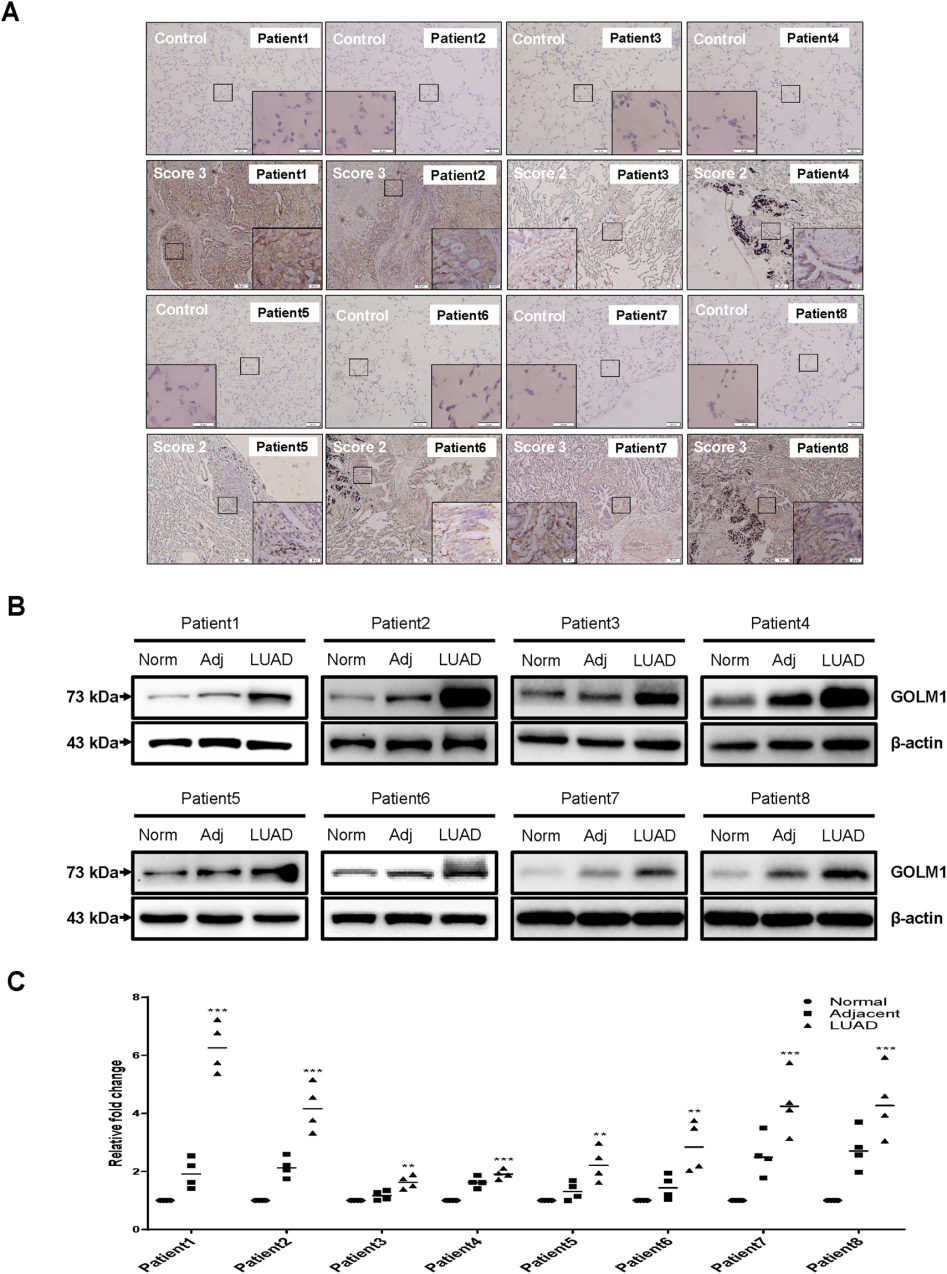
GOLM1 expression is upregulated in lung cancer tissues. **A** GOLM1 expression in clinical samples was measured through immunohistochemical staining. Results were graded by a standardized immunoreactivity score system widely used in clinic. GOLM1 expression of representative images were scored more than two in all clinical samples. **B**, **C** Western blot analysis of GOLM1 protein in clinical samples of eight patients. Num normal tissue, Adj adjacent tissue, LUAD lung adenocarcinoma tissue. Data were means ± SEM. *P < 0.05, **P < 0.01, ***P < 0.001, *n* = 4.

To observe the expression level of GOLM1 in patients with lung cancer, GOLM1 expression in fresh tissue acquired from patients with lung cancer was measured through IHC and western blot. In all eight cases of patients with NSCLC, the expression level of GOLM1 was significantly increased in primary tumor tissues compared with normal tissues (Fig. 2A). As can be seen from the Fig. 2B, it is noteworthy that the expression variations of GOLM1 were relatively high in cancer tissues among different patients with NSCLC. The expression of GOLM1 in patient 1, patient 2, patient 7 and patient 8 significantly increased in tumor samples compared with adjacent samples (*P* < 0.01, Fig. 2C).

### Phosphoproteomics in GOLM1-overexpressing stable PC9 cell lines

**Fig. 3:**
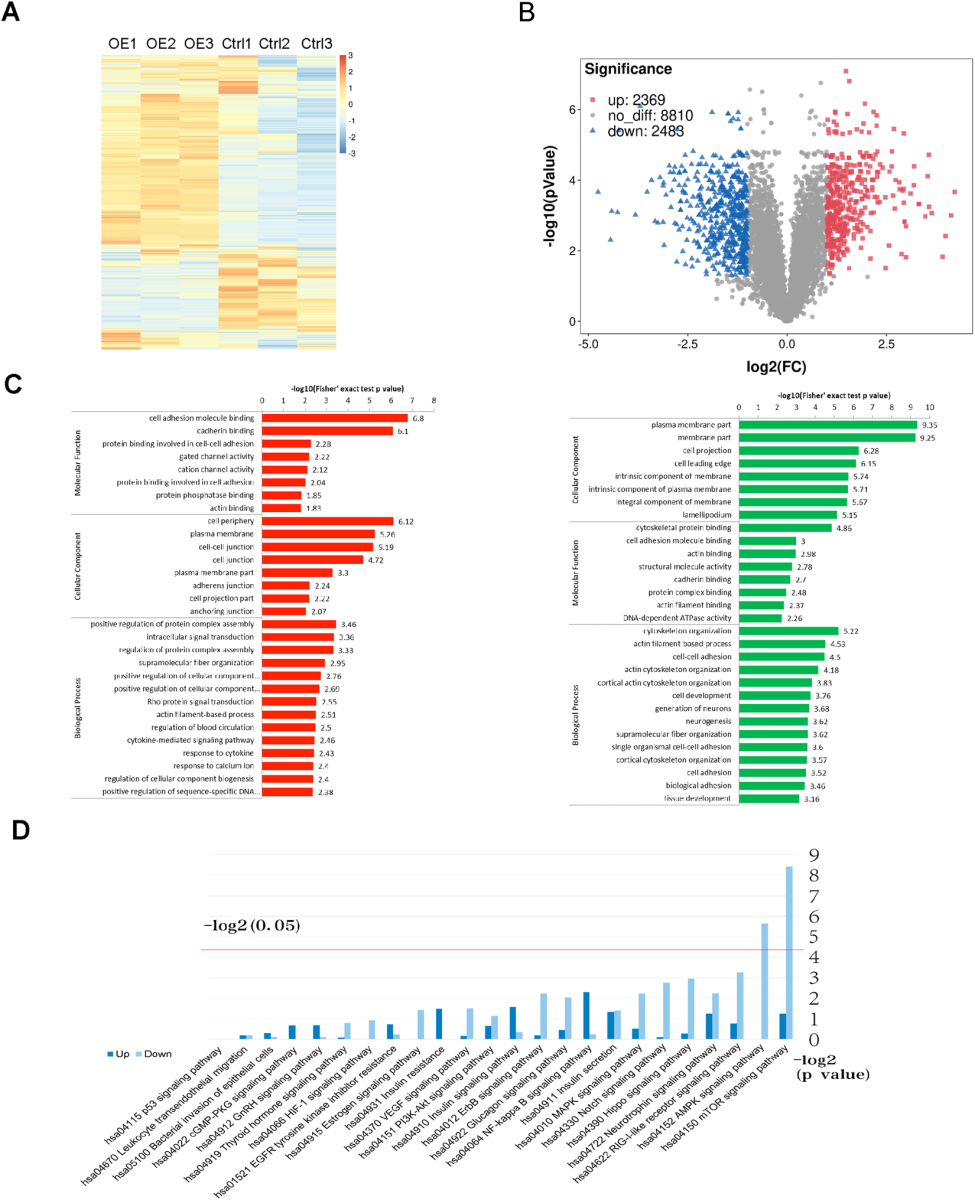
GOLM1-overexpression induced changes in large-scale quantification of the phosphoproteomics. **A** Heatmap of expression levels of differentially phosphorylation modified sites summary between empty vector PC9 cell line and GOLM1 overexpression PC9 cell line. **B** Volcano map of differentially expressed phosphorylating sites between empty vector PC9 cell line and GOLM1 overexpression PC9 cell line (UP refers to proteins with increased phosphorylation in GOLM1OE cells; down refers to proteins with decreased phosphorylation in GOLM1OE cells). **C** GO enrichment results of corresponding proteins at upregulated sites and downregulated sites between empty vector PC9 cell line and GOLM1 overexpression PC9 cell line (left refers to upregulated phosphorylation sites; right refers to downregulated phosphorylation sites). The horizontal axis value is the negative logarithmic conversion of significant *P* value (*P* < 0.05). **D** The KEGG enrichment analysis showed the top 24 signaling pathways.

To investigate the function of GOLM1 in lung cancer, GOLM1-overexpressing stable PC9 cell lines (GOLM1OE PC9 cell lines) were established through lentivirus infection. GOLM1 overexpression in GOLM1OE PC9 cells was identified by western blot analysis (Supplementary Data D). Combining high pressure liquid chromatography (HPLC) with liquid chromatography tandem mass spectrometry (LC–MS/MS), we systematically evaluate the common phosphoproteomics alterations in GOLM1OE PC9 cells. A total of 12,256 independent phosphor-peptides were identified and most of which (8991; 73.36%) containing quantitative information. The heatmap showed the differentially expressed feature of phosphor-peptides between GOLM1OE PC9 cells and empty vector cells (Fig. 3A). We found 2369 upregulated phosphor-peptides and 2488 downregulated phosphor-peptides (Fig. 3B). The average spectral counts for each phosphor-peptide were calculated as 8–20. We synthetically quantified 2709 phosphorylated proteins through analyzing and comparing CDS of identified phosphor-peptides to their corresponding protein sequences in database. To improve the reliability of the phosphoproteomics identification, the identified data were filtered using a standard localization probability >0.75. Finally, the filtered data, including 7816 p-sites on 2594 proteins, were used for subsequent bioinformatics analysis. Furthermore, our data shows that there are 419 p-sites increased and 533 p-sites decreased in their phosphorylation levels respectively between GOLM1-overexpression cells and empty vector cells by comparing the modifications of p-sites (|fold change > 2, *p* value < 0.05).

Correspondingly, we found that 239 and 331 Phosphorylated proteins showed significantly upregulated and downregulated. Top enriched GO terms were selected and categorized into three classes (“Biological Process”, “Cellular Component”, and “Molecular Function”) based on their functional preferences. Comparing WT cells with GOLM1OE PC9 cells, upregulated phosphorylated proteins were more enriched in “Biological Process”, including the classes of “cellular process”, “single-organism process”, and “biological regulation”. When it came to “Cellular Component”, phosphoproteins were more enriched in “cell”, “organelle”, and “membrane”. More importantly, in “Molecular Function”, 210 proteins were enriched in “binding” (Table 1). Meanwhile, downregulated phosphoproteins in “Biological Process” were more enriched in “cellular process”, “biological regulation”, and “single-organism process”, with the number of proteins being 276, 232, and 229, respectively. Regarding “Cellular Component”, Phosphorylated proteins were more enriched in “cell”, “organelle”, and “membrane”, with the number of proteins being 305, 268, and 191, respectively. Furthermore, 298 proteins were enriched in “binding” (Table 2). The phosphoproteins were more enriched in binding-related processes which indicated their potential roles in the regulation of metastasis in lung cancer cells.

**Table 1:** GO secondary annotations of proteins with upregulated p-site (WT vs. OE-GOLM1).

GO terms level 1	GO terms level 2	No. of protein
Biological process	Cellular process	198
	Single-organism process	171
	Biological regulation	170
	Response to stimulus	114
	Metabolic process	107
	Cellular component organization or biogenesis	94
	Signaling	90
	Developmental process	88
	Multicellular organismal process	88
	Localization	82
	Multi-organism process	35
	Immune system process	32
	Locomotion	21
	Biological adhesion	18
	Reproduction	16
	Other	24
Cellular component	Cell	220
	Organelle	182
	Membrane	130
	Macromolecular complex	77
	Membrane-enclosed lumen	74
	Extracellular region	58
	Cell junction	48
	Supramolecular complex	22
	Synapse	12
Molecular function	Binding	210
	Catalytic activity	59
	Molecular function regulator	29
	Structural molecule activity	16
	Transporter activity	16
	Signal transducer activity	15
	Transcription factor activity, protein binding	15
	Nucleic acid binding transcription factor activity	12
	Molecular transducer activity	11
	Other	2

**Table 2:** GO secondary annotations of proteins with downregulated p-site (WT vs. OE-GOLM1).

GO terms level 1	GO terms level 2	No. of protein
Biological process	Cellular process	276
	Biological regulation	232
	Single-organism process	229
	Metabolic process	167
	Cellular component organization or biogenesis	160
	Response to stimulus	148
	Multicellular organismal process	126
	Developmental process	120
	Localization	116
	Signaling	100
	Multi-organism process	56
	Immune system process	48
	Biological adhesion	30
	Locomotion	28
	Reproduction	25
	Other	28
Cellular component	Cell	305
	Organelle	268
	Membrane	191
	Macromolecular complex	110
	Membrane-enclosed lumen	110
	Extracellular region	87
	Cell junction	61
	Supramolecular complex	34
	Synapse	33
	Other	1
Molecular function	Binding	298
	Catalytic activity	95
	Molecular function regulator	32
	Structural molecule activity	28
	Signal transducer activity	22
	Molecular transducer activity	17
	Transcription factor activity, protein binding	17
	Transporter activity	17
	Nucleic acid binding transcription factor activity	16
	Other	3

To estimate malignant biological behavior of cancer cell lines, we then further compared the abundance of phosphorylated proteins within classifications about invasion and migration across WT and GOLM1OE PC9 cells. We found that the clustering of cell adhesion correlations and class of actin cytoskeleton were markedly enriched. Of these, “cell adhesion molecule binding”, “cadherin binding”, “cell periphery”, and “cell–cell junction” in upregulated phosphoproteins were more phosphorylated in GOLM1OE PC9 cells compared with wild-type cells (Fig. 3C). In downregulated phosphorylated proteins, plasma membrane part, cytoskeletal protein binding, and cytoskeleton organization with higher phosphorylation level were in GOLM1OE PC9 cells. The distortion of membrane part, abnormal binding and adhesion events downstream were reconfirmed in lung cancer cell lines showing high malignancy (see Fig. 3C). In KEGG enrichment analysis, the top signaling pathways in downregulated were enriched in mTOR signaling pathway and AMPK signaling pathway. The top signaling pathways in upregulated phosphorylated proteins were enriched in NFkB signaling pathway and insulin signaling pathway (Fig. 3D).

### GOLM1 promotes lung cancer cell proliferation, migration, and invasion

**Fig. 4:**
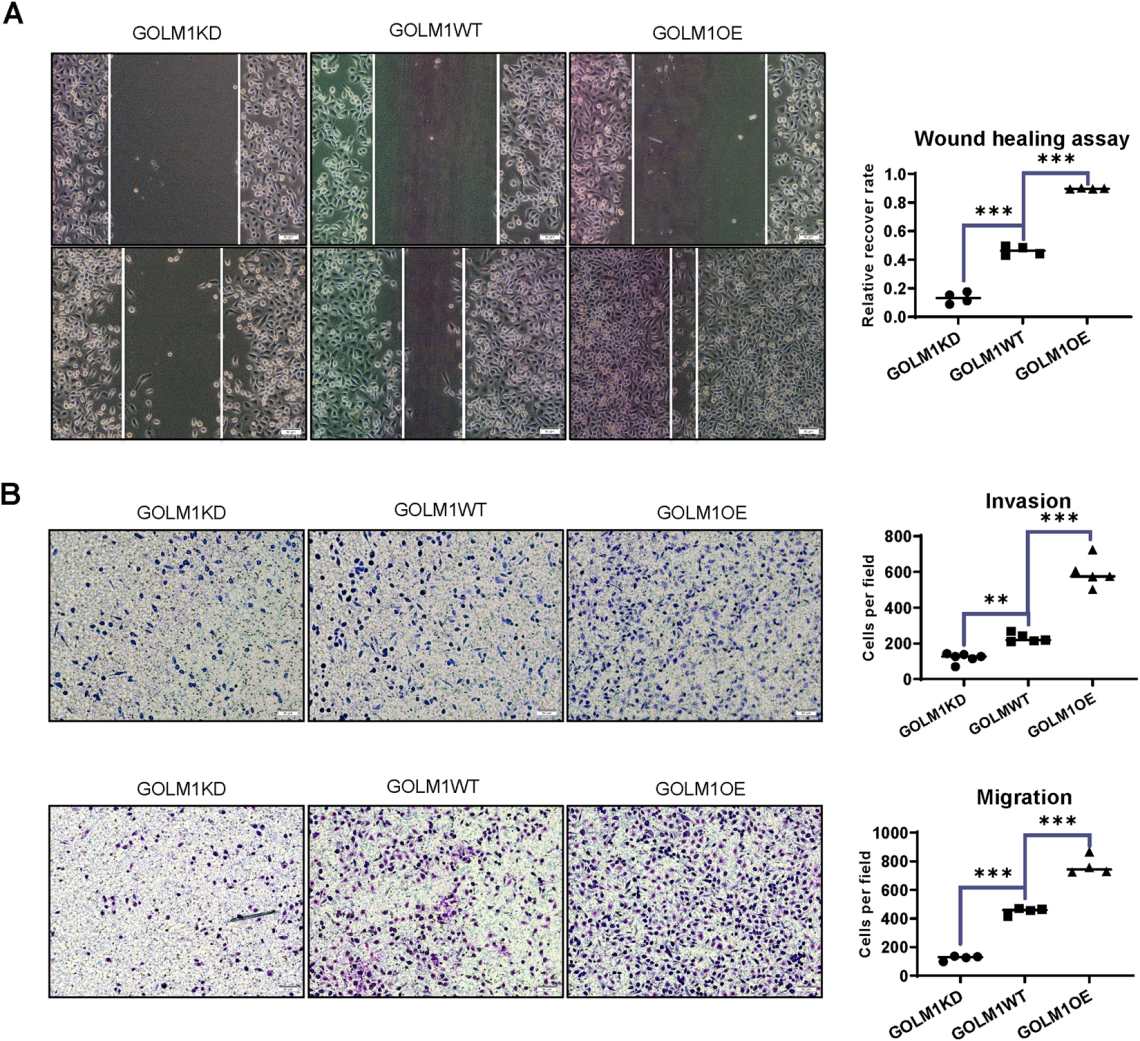
GOLM1 promotes lung cancer cell proliferation, migration, and invasion in vitro. **A** The wound healing ability of different cell lines were evaluated 24 h after seeding. **B** Transwell assays were employed to evaluate the invasion ability and migration ability in different cell lines. GOLM1KD = GOLM1 knockdown group, WT wild-type group, GOLM1OE GOLM1 overexpression group. Data were means ± SEM. *P < 0.05, **P < 0.01, ***P < 0.001, *n* = 3.

To investigate the impact of GOLM1 on cell proliferation and migration, wound healing assays were conducted with the same number of GOLM1OE PC9 cells and GOLM1KD cells. Western blotting checked the expression level of GOLM1 in GOLM1OE PC9 cells and GOLM1KD cells. Compared with wild-type cells, an increased expression of GOLM1 was showed in GOLM1OE PC9 cells, and the expression of GOLM1 was lower in GOLM1KD cells (Supplementary Data C). We observed that GOLM1 overexpression significantly increased the proliferation and migration of cells in GOLM1OE PC9 cells. Knockdown of GOLM1 notably suppressed the proliferation and migration of cells in GOLM1 knockdown cells (Fig. 4A). Similar results were observed through CCK8 assays (Supplementary Data D). Transwell assays were used to observe cell migration and invasion in GOLM1OE PC9 cells and GOLM1KD cells. Knockdown of GOLM1 significantly decreases the migration of PC9 cells. The numbers of migrated PC9 cells were decreased in GOLM1 knockdown cells compared with those in the wild-type cells ((Fig. 4B, *P* < 0.001). However, the numbers of migrated PC9 cells were increased in GOLM1OE PC9 cells compared with those in the wild-type cells (Fig. 4B, *P* < 0.001). Transwell assays also revealed that the extent of invasion was significantly increased in GOLM1OE PC9 cells compared with WT cells while invasive ability of GOLM1KD cells was diminished compared with GOLM1WT cells (Fig. 4B). To rule out the possibility that the differences in invasion and migration may result from the viability between different cell lines, we put them in serum free conditions GOLM1KD, GOLM1WT, and GOLM1OE cells exhibit similar viability through CCK8 assays (Supplementary Data E). These results confirm those that GOLM1 accelerated cell proliferation and migration in lung cancer cell.

### GOLM1 promoted tumor growth in xenograft mouse models of lung cancer

**Fig. 5:**
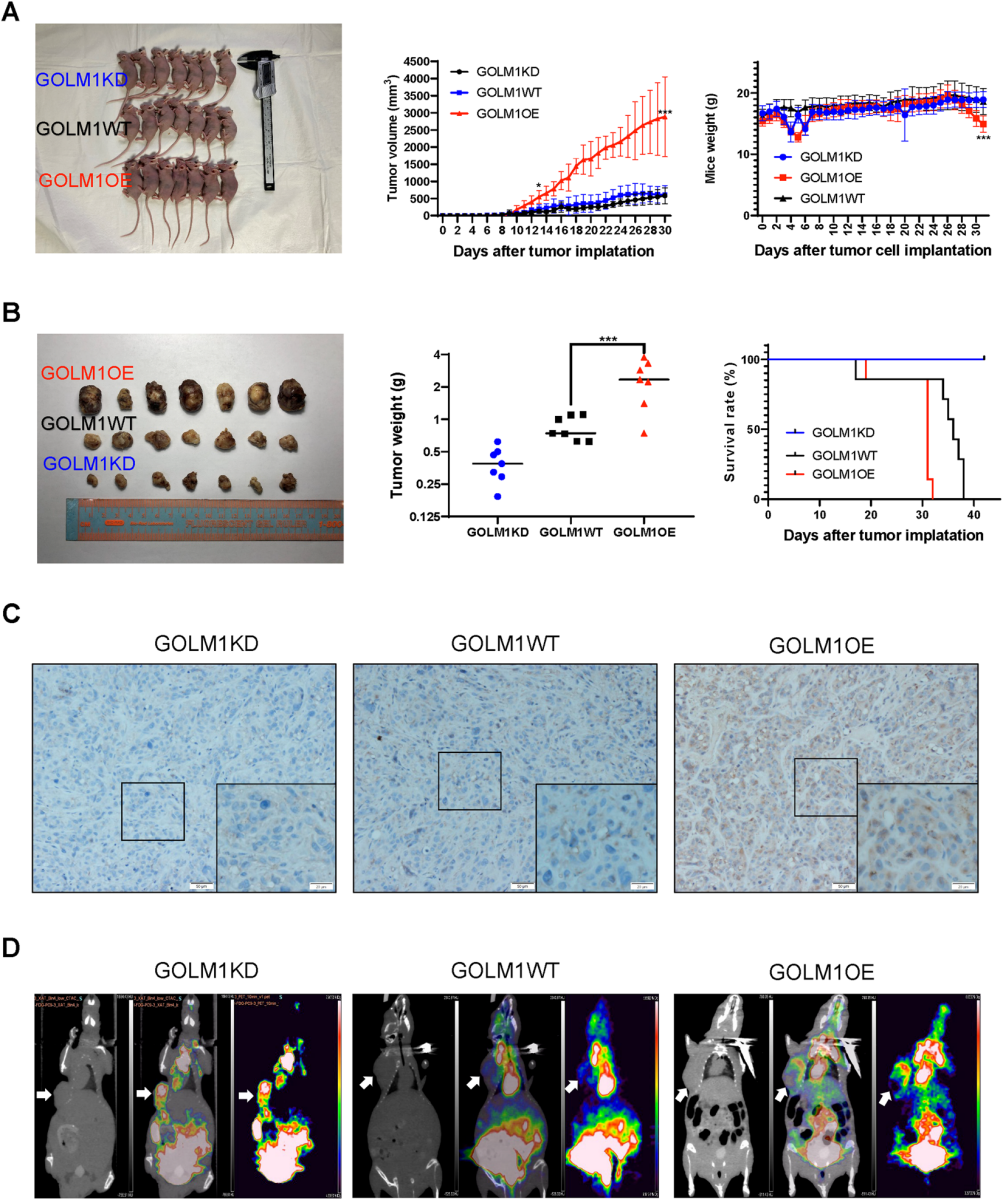
GOLM1 expression is positively correlated with tumor growth in xenograft mice models of lung cancer. **A** Left: Tumors dissected from xenograft mice; middle: weights of tumors in three group; right: the tumor growth curve of xenograft collected from tumor bearing mice. **B** Left: photograph of xenograft mice; middle: weights of xenograft mice in three group; right: the survival curve of xenograft mice. **C** CT, 18F-FDG PET and 18F-FDG-PET/CT images and the maximum standardized uptake value (SUVmax) of xenograft mice. Tumor was pointed with white arrow. GOLM1KD GOLM1 knockdown group, WT wild-type group, GOLM1OE GOLM1 overexpression group. Data were means ± SEM. *P < 0.05, **P < 0.01, ***P < 0.001, *n* = 3.

Tumor xenograft animal models for GOLM1 research are developed by subcutaneously implanting GOLM1OE PC9 cells or wild-type PC9 cells in nude mice. After 10 days, the mean tumor volume increased rapidly in GOLM1 overexpression xenograft mice compared with wild-type control mice (Fig. 5A, left). We found that the tumor size in mice injected with GOLM1OE cells were significantly increased than those injected with GOLM1WT cells (Fig. 5A, middle). We observed significant differences in weights of xenograft mice at final stage in two groups of tumor-bearing mice. The final weights of nude mice subcutaneously implanted with GOLM1OE cells were significantly lower than those of control mice (Fig. 5A, right). At the end of the experiment, the subcutaneous tumors were removed (Fig. 5B, left). The tumor weights of mice injected with GOLM1OE cells were significantly increased than those injected with GOLM1WT cells (Fig. 5B, middle). Tumor xenograft for GOLM1OE cells decreased the survival time compared with control mice (Fig. 5B, right). Tumor xenograft for GOLM1OE cells exhibited an increased GOLM1 expression in tumor compared with control mice (Fig. 5C). To accurately evaluate the growth of tumors, micro positron emission tomography/computed tomography (micro-PET/CT) was used to image tumor-bearing mice using 2–18F-fluoro-2-deoxy-d-glucose (FDG; Fig. 5D). The images of the conjugate of CT and PET showed that FDG uptakes in tumors were easily visualized in axial images at the 1-h time point after injection. For GOLM1 overexpression, uptake was relatively less compared with WT which causing by necrosis of the tumor center. Our results further demonstrated that GOLM1 overexpression could significantly increase the growth of tumor in vivo.

### The overexpression of GOLM1impact the p53 tetramer

**Fig. 6:**
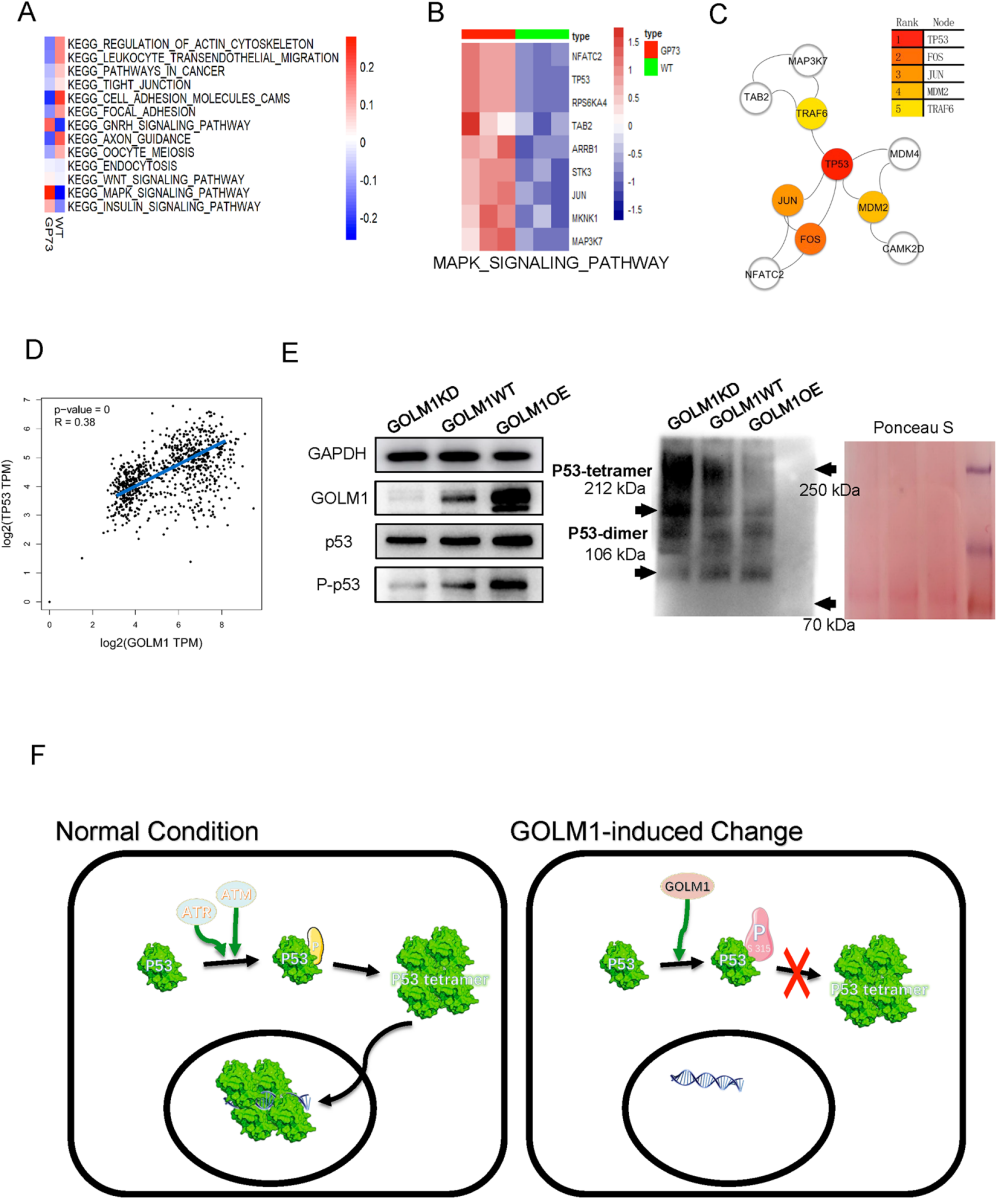
GSEA reveals that GOLM1 promotes growth of lung cancer cells via regulating tetramer formation of P53. **A** GSEA results of KEGG pathway cluster analysis from phosphoproteomics data. **B** The expression level of members in MAPK signaling pathway enriching from GSEA result. **C** Protein–protein interaction (PPI) of hub genes in MAPK signaling pathway. **D** Pair-wise gene expression correlation analysis between GOLM1 and TP53. **E** Western blot results of expression level of P53, S315 Phos-P53 protein, and P53 tetramer protein in three groups from GOLM1KD, GOLM1WT, and GOLM1 OE PC9 cells (GOLM1, P53, S315 Phos-P53 protein were separated by SDS-PAGE; P53 tetramer (212 kDa) and P53 dimer (106 kDa) protein were separated by native-PAGE). **F** Mechanism of GOLM1 promotes growth of lung cancer cells through regulating tetramer formation of P53. GOLM1KD GOLM1 knockdown group, GOLM1WT wild-type group, GOLM1OE GOLM1 overexpression group.

To reveal the impact of GOLM1 on various pathways in lung cancer, GSEA analysis were performed to interpret the feature gene sets in each subtype. Subtype various pathways are exhibited by different color. MAPK signaling pathway was identified as highly correlated with GOLM1 overexpressing (Fig. 6A). These expressions of genes enriched in MAPK signaling pathway in GOLM1 overexpressing cells were found to increase compared with WT cell (Fig. 6B). These high expressions of protein enriched in MAPK signaling pathway were input into the STRING tool, and then the resulting data were imported into Cytoscape. P53 was mapped as Hub genes through cytoHubba plug-ins (Fig. 6C). To confirm the effect of GOLM1 in P53, we used GEPIA to draw scatter plots based on TCGA data (Fig. 6D), which demonstrates a medium correlation between P53 and GOLM1 (*R* = 0.38). To verify this finding, the dataset from TCGA database contains 496 patients with NSCLC which were grouped according to P53 mutation (*n* = 252) or not (*n* = 244), were downloaded, collated, and analyzed. Each group was then further grouped according to the expression level of GOLM1 and draw a survival curve. We found that there was no correlation between the expression of GOLM1 and the survival rate in NSCLC patients with P53 mutation (*p* = 0.06252), but a significant correlation was found in NSCLC patients expressing wild-type p53 (*p* = 0.02449) (see Supplementary Data F). To further confirm that GOLM1 exercises oncogenic role dependent on P53 involvement we hired P53 inhibitor (PFT-α) and activator (Nutlin3). We observed a significant inhibitory effect of P53 activator (Nutlin3) on GOLM1OE cell survival (see Supplementary Data G), while P53 inhibitor (PFT-α) have been shown to increase GOLM1KD cell survival (see Supplementary Data H). Through western blot, we verified that the phosphorylation of P53 protein at site S315 was enhanced in the GOLM1 overexpressing group (GOLM1OE) and decreased in the GOLM1 knockdown group (GOLM1KD). More importantly, we also demonstrated that P53 tetramers in the GOLM1 overexpression group were significantly less than those in the control group, while GOLM1 knockdown group was significantly higher than that in the control group (Fig. 6E). Figure 6F illustrate a model delineating the role of GOLM1 in lung cancer. GOLM1 overexpression increased the phosphorylation of P53 protein at site S315, but it inhibited forms of P53 tetramers.

## Discussion

From research on the role it plays in cancer^12,13^, GOLM1 is considered a unique biomarker in the diagnosis of many kinds of malignant diseases and as a candidate of targets for targeted therapy. However, for lung cancer, we know little about what the function of GOLM1. In this study, we showed that GOLM1 may have a vital role in the progression of lung cancer because we observed the overexpression of GOLM1 in all tumor tissues acquired from surgery. By manipulating the expression of GOLM1 and conducting assays, we found GOLM1 promoted lung cancer cell proliferation, migration, and invasion. Furthermore, in in vivo studies, increased tumor growth in a xenograft mouse model of lung cancer occurred after over-expressing of GOLM1 in inoculated cells.

GOLM1 encodes an epithelial-specific Golgi membrane protein, a single-pass type II membrane protein, which is expressed in various tissues^17^. The early literature indicated that GOLM1 was discovered highly expressed in the colon, trachea, stomach and prostate, and to a lesser extent in testes, muscle, lymphoid tissues, white blood cells and the spleen^18^. More importantly, GOLM1 is highly expressed by cells of the epithelial lineage^19^. GOLM1 participates in mitosis, invasion, and the migration of cancer cells as a key members of Golgi in which posttranslational proteins being modified and sorted^20^. Our effort in exploring networks adjusting functions of Golgi apparatus that affect the biological behavior of cancer cells may pave the way to uncover a mechanism for malignancy progression. High correlation between lung cancer and upregulation of GOLM1 expression were confirmed in this study.

The mechanisms by which GOLM1 is involved in lung cancer have not been clarified. We can draw lessons from discussions on other cancer types. For example, researchers from Fudan University found that GOLM1 promoted the metastasis of hepatocellular carcinoma cells by regulating the redistribution of EGFR/RTK in the cell cycle^13^. A current study has revealed that alterations in DNA copy number and methylation may be two important mechanisms used a dysregulated GOLM1 in lung adenocarcinoma^15^. In our study, we focused on uncovering the functions of GOLM1 in lung cancer. We constructed a GOLM1-overexpressing cell lines from PC9 cells (OE-GOLM1), and silenced endogenous GOLM1 expression by lentivirus in PC9 cells (shGOLM1). Unlike previous studies, we sought to find out the effect of GOLM1 on cellular function at protein levels. Protein is the basic unit for performing cell function. Activation of the phosphorylation pathway is an important molecular and pathological mechanism of lung cancer which plays an important role in tumorigenesis, progression, and metastasis. Therefore, we have used phosphorylated proteins of cells as research objects. We employed a global phosphoproteomics approach to discover protein phosphorylation profiles in lung cancer cell lines with different expression levels of GOLM1. We also identified and quantified thousands of phosphorylated proteins, gaining an insight into a phosphoproteomics view in cancer biology from a macro perspective.

Through comparing the expression level of phosphorylation sites between OE-GOLM1 and WT groups, several protein domains and pathways promoting aberrant biological behavior of cancer cells were identified. Cluster analysis indicated that GOLM1 overexpression altered the “actin cytoskeleton”, which has been related to more aggressive subtypes of cancer cells in other researches^21^. Furthermore, changes relating to “adhesion” and binding”, which are more enriched in GOLM1 overexpression, correlated highly with cancer cell invasion and migration. This correlates with the viewpoint that adjusting the expression of cell adhesion molecules may alter the cross-talk between cells and microenvironment which suggests that such specific processes deeply involve in tumor progression through influencing cell invasion and metastasis^22^. Following the cell scratch test, transwell cell invasion assay and transwell cell migration assay, we proved overexpression of GOLM1 facilitated migration and invasion of lung cancer cells in vitro. Furthermore, by establishment of tumor-bearing mice model, we witness tumor growth more seriously GOLM1 promotes lung cancer tumor growth in vivo. To sum up, combined our cellular assays and animal trials, we demonstrated that overexpression of GOLM1 facilitating lung cancer progression as an indispensable factor.

The MAPK signaling pathway functions as a tumor-suppressor or pro-oncogenic signal, which is regulated by phosphorylation mechanisms that bidirectionally communicates with the PI3K/AKT signaling pathway; information on the cell cycle, proliferation progression and apoptosis coalesces at this crucial node. The marked changes in phosphorylation profiles of key points in the MAPK pathway may hint at its activation, together with PI3K/AKT, in more aggressive cancer cell lines. GSEA results showed that among the differentially expressed genes in MAPK signal pathways, TP53 was located at the central position of all the hub genes and ranked first of them. P53, as a tumor suppressor gene, plays an important role in maintaining cell homeostasis. The phosphorylation of p53 is an important step to stabilize P53 and enhance its transcriptional activity. The phosphorylation of P53 is a process in which phosphate groups are added to the side chain hydroxyl group of an amino acid residue of p53 protein. The common amino acids include serine (Ser) and threonine (Thr) residues. P53 S315 and S392 are the earliest identified phosphorylation sites at the c-terminal. Previous researches have revealed that phosphorylation at the ser392 site stabilizes p53 tetramers’ formation^23,24^. On the contrary, S392’s phosphorylation was reversed by S315’s phosphorylation on tetramer’s formation and the stable activation of p53^25,26^. The phosphorylation at S315 inactivates P53 by enhancing its protein degradation^27,28^. In this study, the hyperphosphorylation of S315 in P53 in GOLM1-overexpressing cells was noticed comparing with WT cells which may deeply affect the formation of the P53 tetramer and further weak P53 mediated inhibition of tumor formation. In other words, GOLM1 overexpression enhances the aggressive malignant manner of cancer cells by reducing the stability of P53 (Fig. 5G). Our future work will focus on the specific mechanism by which GOLM1 affects the stability of P53.

## Conclusion

GOLM1 as a critical oncogene facilitates lung cancer cell proliferation, migration and invasion. GOLM1 has oncogenic functions affecting many signaling pathways. It can be used as a stable biomarker for screening patients who may have a relatively higher risk of cancer. More specific exploration of its molecular mechanisms in the progression of lung cancer is needed in future studies. Therefore, agents that affect GOLM1 directly or downstream of GOLM1 activity can potentially be used in patients with lung cancers that show high expression of GOLM1.

## Supplementary information

Supplementary data A

Supplementary data B

Supplementary data C

Supplementary data D

Supplementary data E

Supplementary data F

Supplementary data G

Supplementary data H
