# Supplementary figures and images for: The functional landscape of Golgi membrane protein 1 (GOLM1) phosphoproteome reveal GOLM1 regulating P53 that promotes malignancy
Source: Cell Death Discov. 2021 Mar 1;7:42. doi: 10.1038/s41420-021-00422-2 (PMC7921442; doi:10.1038/s41420-021-00422-2)

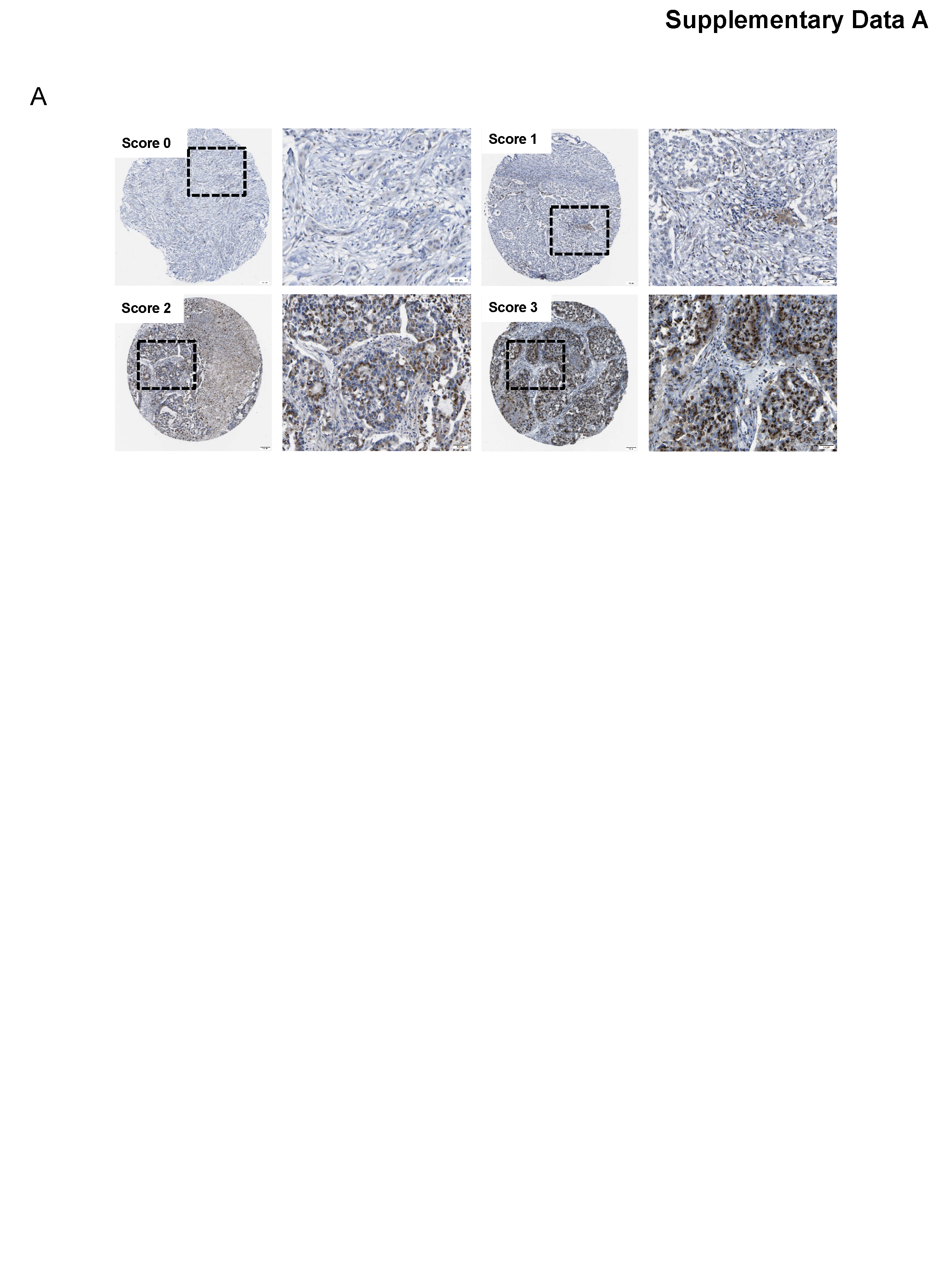

Supplement: Supplementary file 1 — Supplementary data A [file 41420_2021_422_MOESM1_ESM.tif]

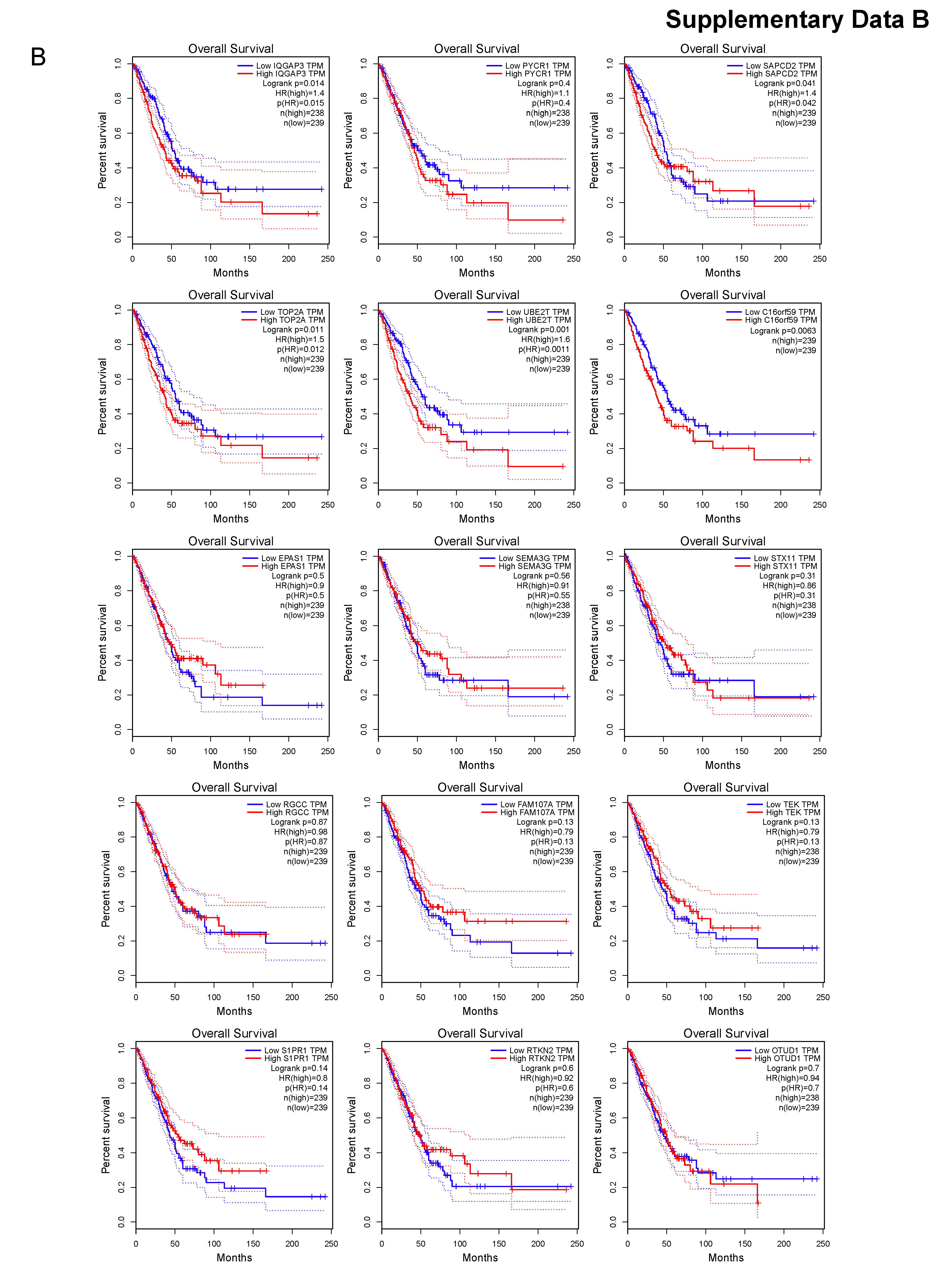

Supplement: Supplementary file 2 — Supplementary data B [file 41420_2021_422_MOESM2_ESM.tif]

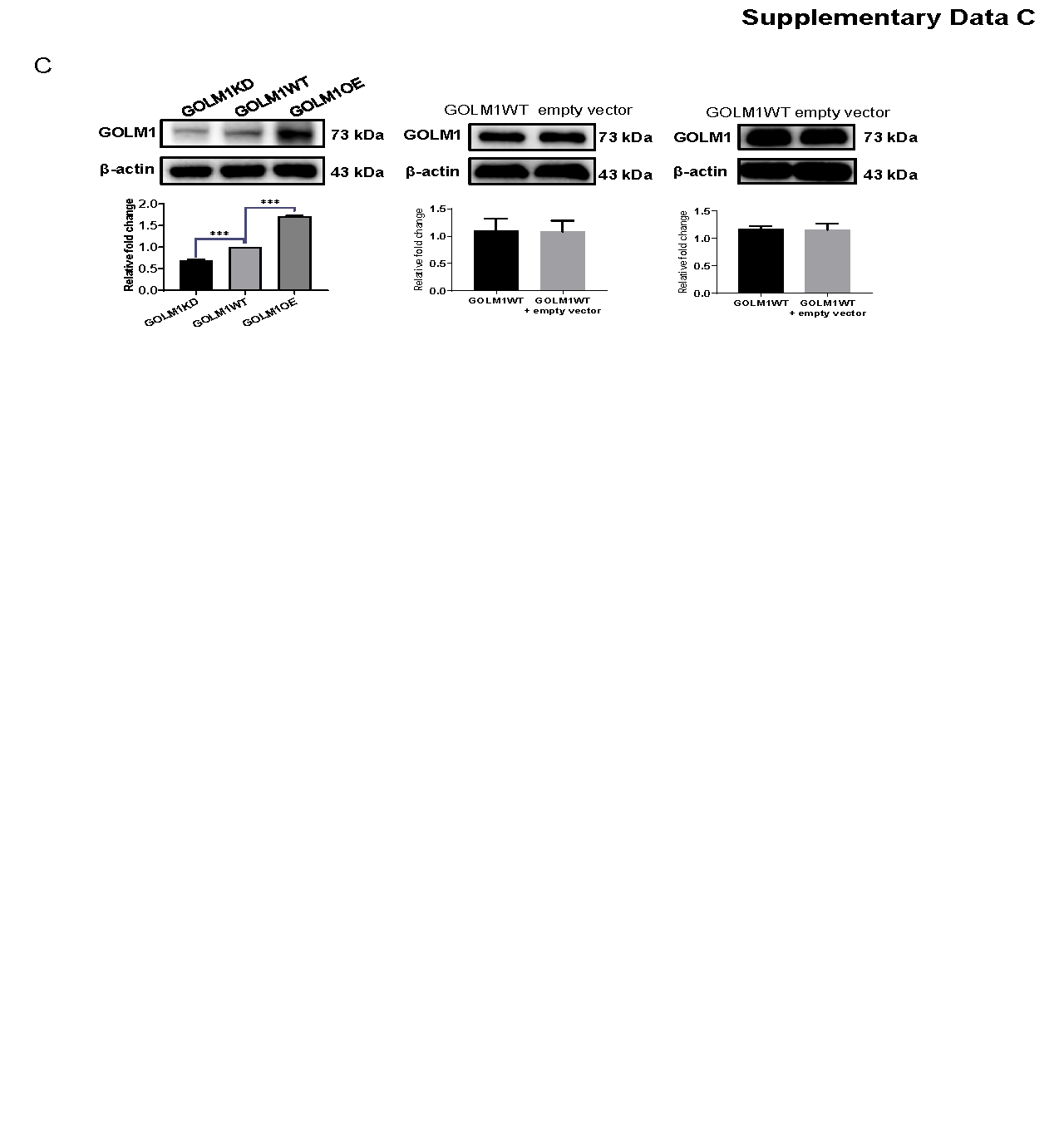

Supplement: Supplementary file 3 — Supplementary data C [file 41420_2021_422_MOESM3_ESM.tif]

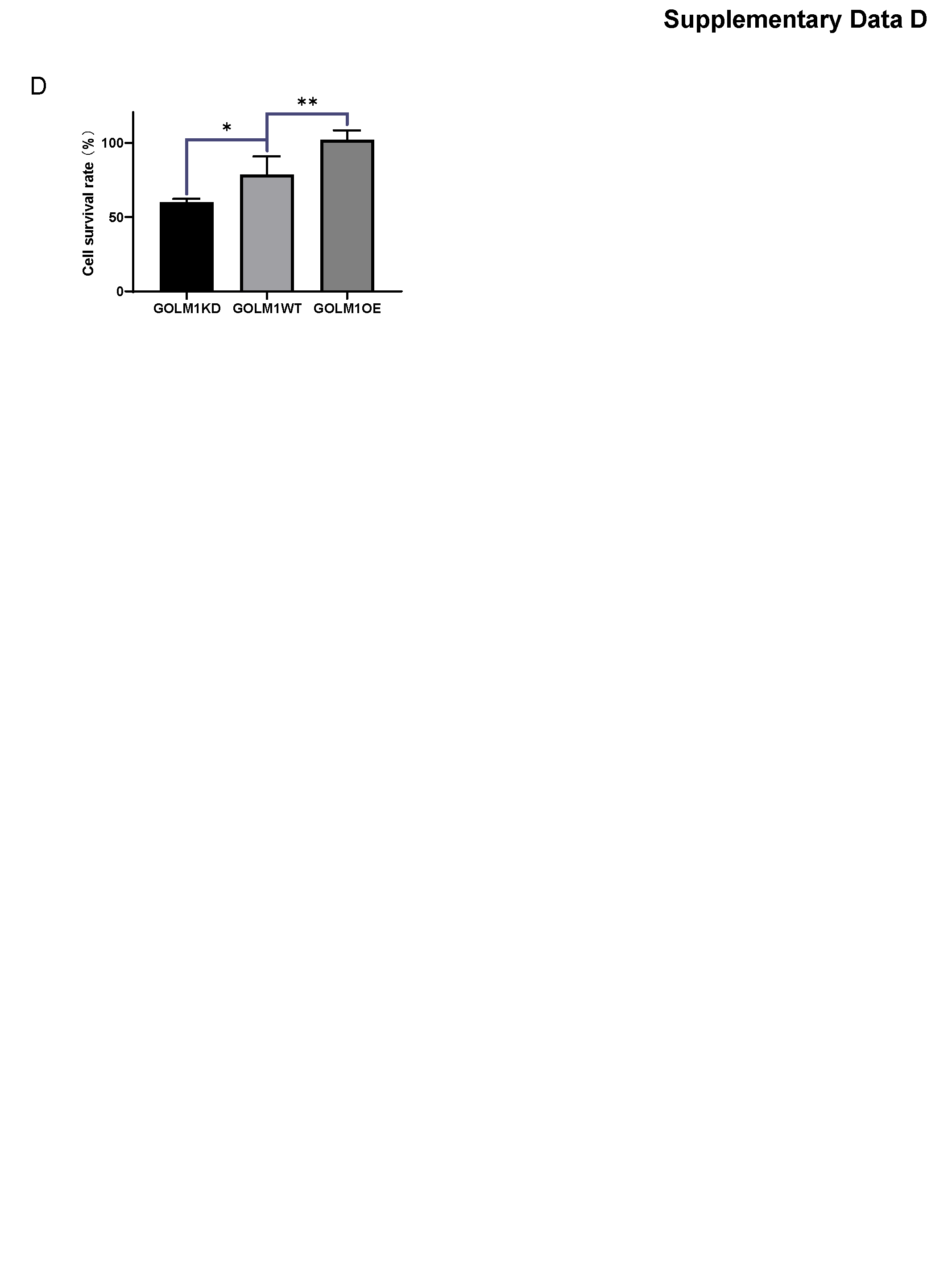

Supplement: Supplementary file 4 — Supplementary data D [file 41420_2021_422_MOESM4_ESM.tif]

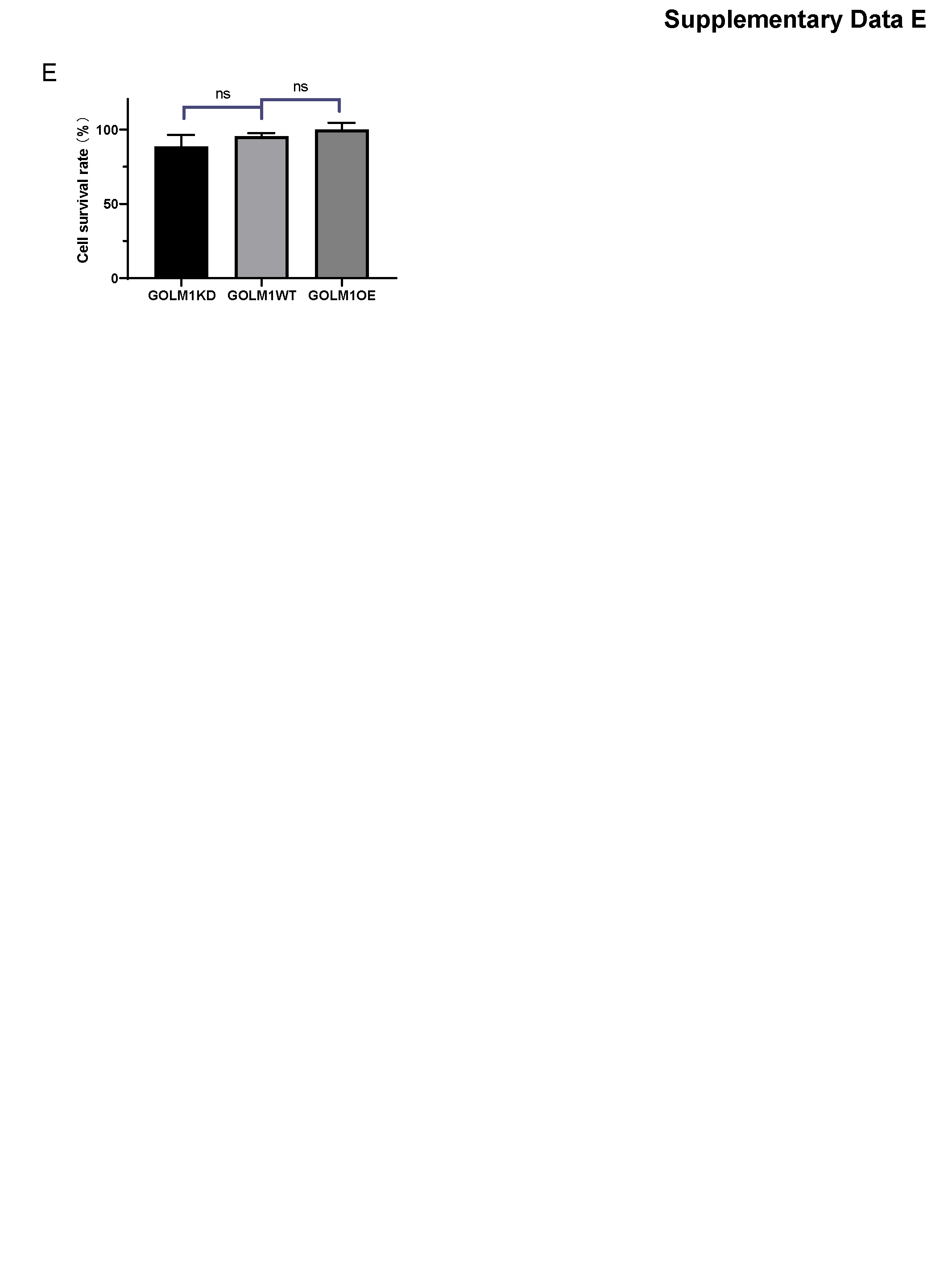

Supplement: Supplementary file 5 — Supplementary data E [file 41420_2021_422_MOESM5_ESM.tif]

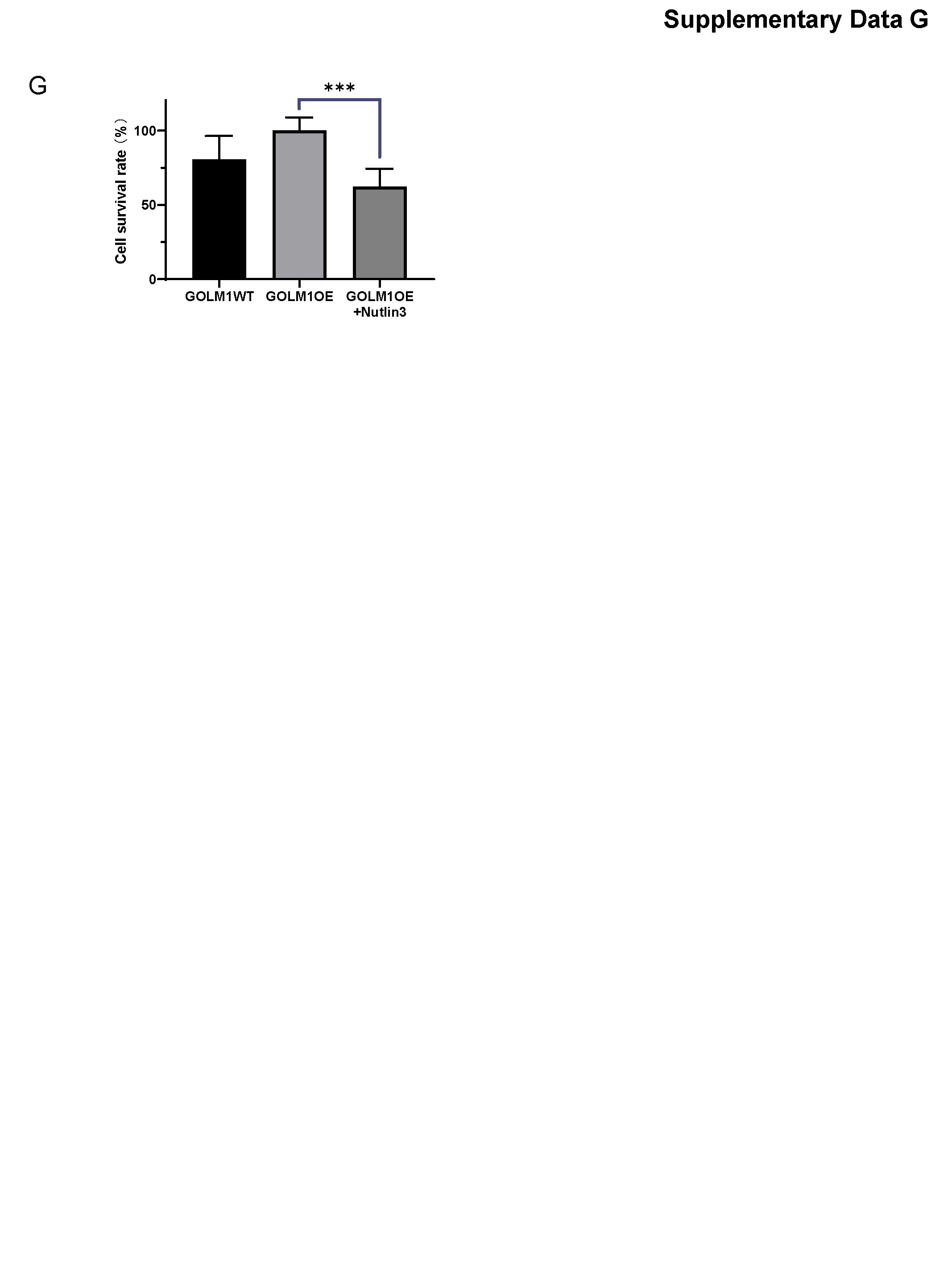

Supplement: Supplementary file 7 — Supplementary data G [file 41420_2021_422_MOESM7_ESM.tif]

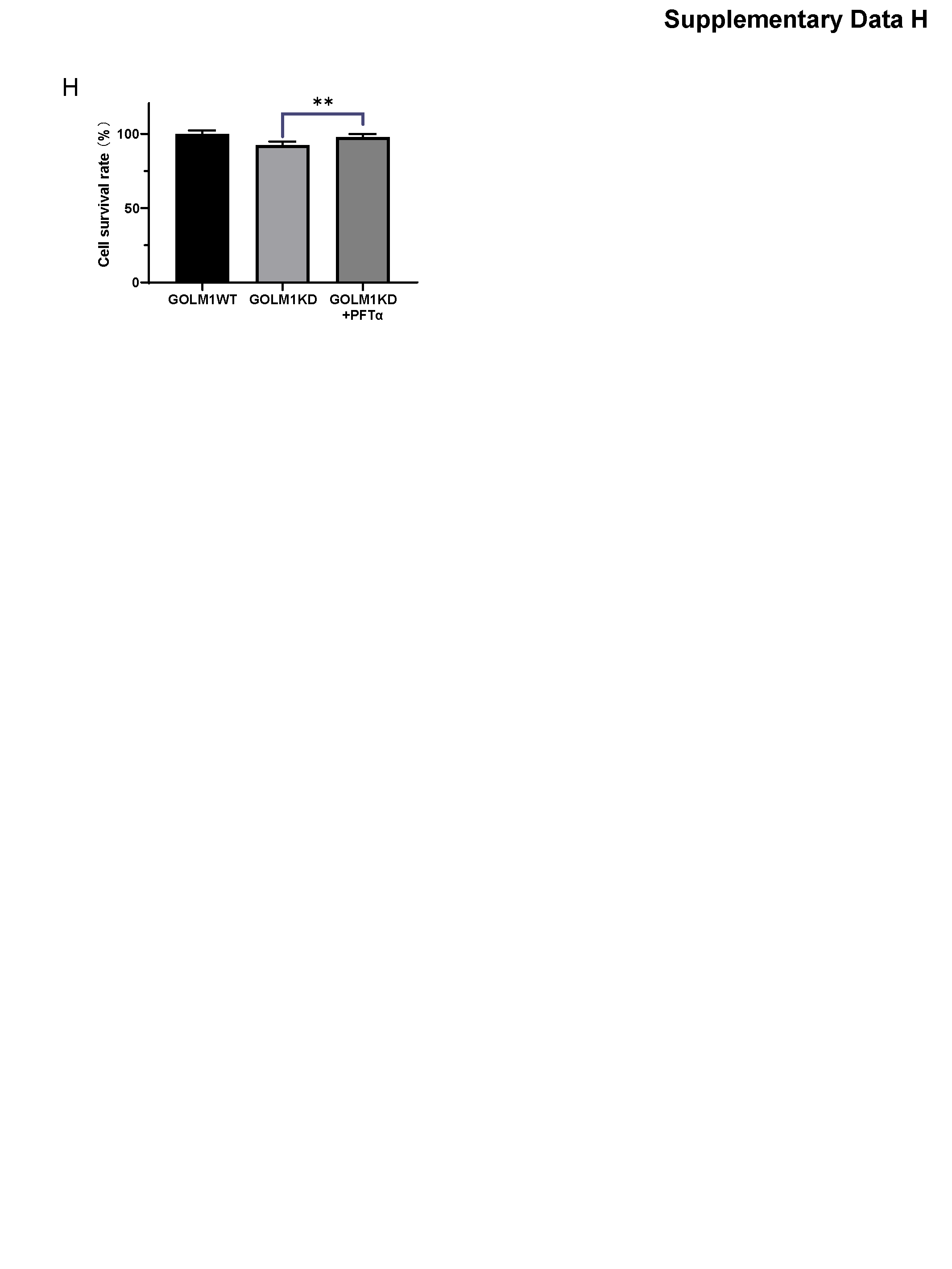

Supplement: Supplementary file 8 — Supplementary data H [file 41420_2021_422_MOESM8_ESM.tif]
